# A Molecular Switch Driving Inactivation in the Cardiac K^+^ Channel hERG

**DOI:** 10.1371/journal.pone.0041023

**Published:** 2012-07-24

**Authors:** David A. Köpfer, Ulrike Hahn, Iris Ohmert, Gert Vriend, Olaf Pongs, Bert L. de Groot, Ulrich Zachariae

**Affiliations:** 1 Computational Biomolecular Dynamics Group, Max Planck Institute for Biophysical Chemistry, Göttingen, Germany; 2 Center for Molecular Neurobiology, Institute for Neural Signal Transduction, University of Hamburg, Hamburg, Germany; 3 Center for Molecular and Biomolecular Informatics, Radboud University Nijmegen Medical Center, Nijmegen, The Netherlands; 4 Scottish Universities Physics Alliance, School of Physics and Astronomy, The University of Edinburgh, Edinburgh, United Kingdom; Georgia State University, United States of America

## Abstract

K^+^ channels control transmembrane action potentials by gating open or closed in response to external stimuli. Inactivation gating, involving a conformational change at the K^+^ selectivity filter, has recently been recognized as a major K^+^ channel regulatory mechanism. In the K^+^ channel hERG, inactivation controls the length of the human cardiac action potential. Mutations impairing hERG inactivation cause life-threatening cardiac arrhythmia, which also occur as undesired side effects of drugs. In this paper, we report atomistic molecular dynamics simulations, complemented by mutational and electrophysiological studies, which suggest that the selectivity filter adopts a collapsed conformation in the inactivated state of hERG. The selectivity filter is gated by an intricate hydrogen bond network around residues S620 and N629. Mutations of this hydrogen bond network are shown to cause inactivation deficiency in electrophysiological measurements. In addition, drug-related conformational changes around the central cavity and pore helix provide a functional mechanism for newly discovered hERG activators.

## Introduction

Regulated current through K^+^ channels plays an essential role in cellular ionic homeostasis and intercellular signaling [1]. Although activation gating – a large-scale reconfiguration of the pore-forming transmembrane helices – had long been viewed as the main regulatory switch of K^+^ channels, C-type inactivation and the coupling between activation and inactivation have recently been recognized as general control mechanisms of K^+^ channel gating [2]–[6]. There is increasing evidence that the inactivation gate of K^+^ channels resides near the K^+^ selectivity filter (SF), and that C-type inactivation entails a conformational change of the filter itself [3]–[5], [7]–[9]. C-type inactivation plays a particularly important role in the K^+^ channel hERG (human ether-a-go-go related gene potassium channel, Kv11.1).

**Figure 1:**
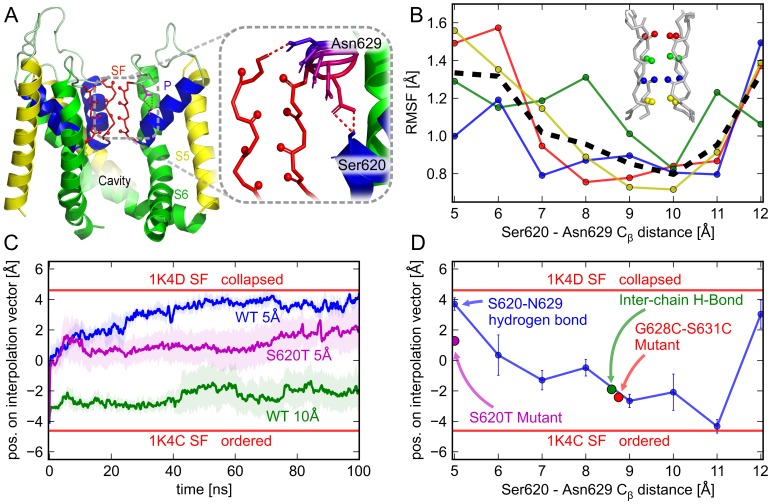
Model structure of the hERG channel and switch behind the selectivity filter. (**A**) Model of the hERG channel, lined by the S6 helices (green), and including the K^+^ selectivity filter (SF, red), pore helices (P, blue), internal cavity and outer pore loop. As structural information on the turret loops is sparse and modeling according to homology is not possible in this region, the loops were modeled as in KvAP [28]. (A, inset) Scan of H-bonds between N629 and S620. (**B**) Dependence of the backbone carbonyl fluctuation (RMSF) of SF residues S624–F627 on the distance between S620 and N629. (**C**) Small separations between S620 and N629 (5 Å, blue curve) promote SF collapse (1K4D, upper red bar), while larger separations (10 Å, green curve) stabilize its conductive state (1K4C, lower red bar). For an equally short separation (5 Å, magenta curve) the S620T mutant displays a marked deviation from the WT. Each time trace represents the mean of four independent simulations with their standard error (shaded area) (**D**) Direct dependence of the extent of SF collapse on the interaction between N629 and S620 (blue line). The formation of a stable inter-chain H-bond to G628 stabilizes the conductive SF (green circle). The non-inactivating mutant S620T does not reach a fully collapsed state even at a T620-N629 distance of 5 Å (magenta circle), while the double mutation G628C/S631C precludes a close contact between N629 and S620 and hence a transition to the collapsed state (red circle).

hERG is a channel protein predominantly expressed in human cardiac myocyte membranes [10], [11]. It forms a pore at the interface of four subunits each containing six transmembrane (TM) helices and the pore helix. The pore comprises the K^+^ selectivity filter (SF) and a central, water-filled cavity (Fig. 1A) [10], lined by the innermost TM helices S5 and S6. In addition to its cardiac function, hERG also appears to contribute to tumor cell proliferation and apoptosis [12]. In most K^+^ channels, C-type inactivation is a slow process that decreases channel current on time scales of seconds. In hERG, it proceeds much faster (0.7–2.9 ms) and thus dominates its conductance properties. As the repolarization phase of the human cardiac action potential is governed by flux through hERG, its kinetics determine the length of the action potential and, thereby, strongly contribute to normal function of the heart [10], [13].

hERG malfunction is thus implicated in many forms of cardiac arrhythmia, which affect up to 1 in 5000 humans and are a common cause for sudden death [14]–[16]. The highest arrhythmic risk is associated with hERG mutations in the pore region which affect inactivation, and with undesired drug binding to hERG, again primarily affecting the inactivated form of the channel [17]–[22]. Long-QT syndrome is caused by loss of hERG function, either through misfolding, trafficking defects, or hERG missense mutations, while impairment of inactivation induces short-QT syndrome via gain of hERG function [23]–[26].

To understand inherited or acquired short- and long-QT syndrome, insights into the mechanistic basis for inactivation gating are essential. It has been suggested that the inactivated state of the hERG SF resembles the collapsed (low-[K^+^]) configuration of the SF (as displayed by the crystal structure of KcsA; [27]), but this hypothesis awaits validation [22]. We used our recently developed consensus structure model of the hERG open state [28] to investigate the driving forces, nature, and consequences of the conformational change that leads to hERG inactivation. This model has recently been shown to quantitatively reproduce experimental hERG blocker structure-activity relationships [29]. Combinations of in silico molecular dynamics (MD) and docking studies with in vitro and in vivo mutagenesis and electrophysiology studies revealed the pathway of the conformational change at the SF and a distinct molecular switch that toggles the SF between conductive and collapsed states.

## Results

### Tightening of a Hydrogen Bond Induces Collapse of the hERG Selectivity Filter

Crystal structural and electrophysiological studies on KcsA have indicated that hydrogen bonding between residues E71 and D80 behind the SF affects C-type inactivation in this prokaryotic K^+^ channel [8], [9]. Inactivation is thought to involve conformational changes within a network of residues encompassing the SF as highly conserved structural element [30]. Many mutations that affect hERG inactivation scatter around the homologous hERG sequence positions S620 and N629 as central residues [18]. According to most present hERG structural models, these residues are arranged behind the fully conserved hERG SF [31]–[33], however without a close and direct interaction in most cases [32], [33]. On the basis of our recent consensus structural model [28], we carried out a computational interaction scan, in which the distance between the side chains of S620 and N629 was systematically varied in intervals of 1 Å (Fig. 1A). We then monitored the response of the inner SF, i.e. the backbone region between residues S624 and G627, in extensive MD simulations. The most pronounced level of conformational variability was observed in simulations with a transiently vacated SF. To distinguish protein-mechanistic from ion-induced effects, we repeated the simulations with varying ion occupancy in the SF (Fig. S1).

As shown in Fig. 1, the configuration of S620 and N629 markedly influenced the dynamics of the SF. The root mean square fluctuation (RMSF) of the carbonyl oxygen atoms that coordinate K^+^ ions at the binding sites S1–S4 [27] was minimal when the distance between S620 C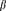 and N629 C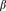 remained close to 10 Å (Fig. 1B). Especially the fluctuation of the terminal groups of oxygen atoms in the SF (S624 and F627) was modulated by up to a factor of 2 by the distance between S620 and N629. The average fluctuation levels varied between 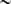
0.8 Å and 1.6 Å, depending on their separation. At distances larger than d = 11 Å, a sharp increase in fluctuation was seen owing to stretching of the SF backbone.

To investigate the structural consequences of such increased flexibility, we examined hERG configurations with minimum and maximum fluctuation levels. The obtained SF conformations were then compared to high-resolution crystallographic data. We chose the highest-resolution structures of the KcsA SF backbone in their high-[K^+^] (conductive, PDB ID 1K4C; [27]) and low-[K^+^] (collapsed, PDB ID 1K4D; [27]) configurations as comparison, and used the projection of the SF configurations on the difference vector between these extreme geometries as reaction coordinate.

Fig. 1C shows that a conformational change from the conductive (1K4C) to a collapsed SF state (1K4D, horizontal red bars) was elicited when the interaction distance between S620 and N629 was closest (d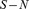 = 5 Å, measured between the respective C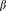 atoms in four independent simulations, compatible with an intact H-bond, blue curve). The conformational change occurred on a time scale of 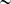
40 ns. In sharp contrast, a wider S620–N629 separation (d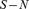 = 10 Å) stabilized the highly ordered, conductive state of the SF (Fig. 1C, green curve), even without the presence of ions in the filter. For comparison, in KcsA, collapse of the SF to its low-[K^+^] state has recently been identified as the most likely cause of slow inactivation [3]–[5]. In addition, hydrogen bonding between the residues homologous to S620 and N629 in KcsA, E71 and D80, has been shown to directly influence entry into the C-type inactivated state of KcsA [8], [9]. It has been debated whether the mechanism of hERG inactivation may be fundamentally different from other K^+^ channels, in particular from slow inactivation in KcsA [30]. For instance, there are major differences in the response to extracellular tetraethylammonium and K^+^ concentration. However, recent mechanistic insights gained by mutation studies and simulations point toward many common mechanistic features shared by SF inactivation in the pore domain of most K^+^ channels, including KcsA and hERG [4], [30].

**Figure 2:**
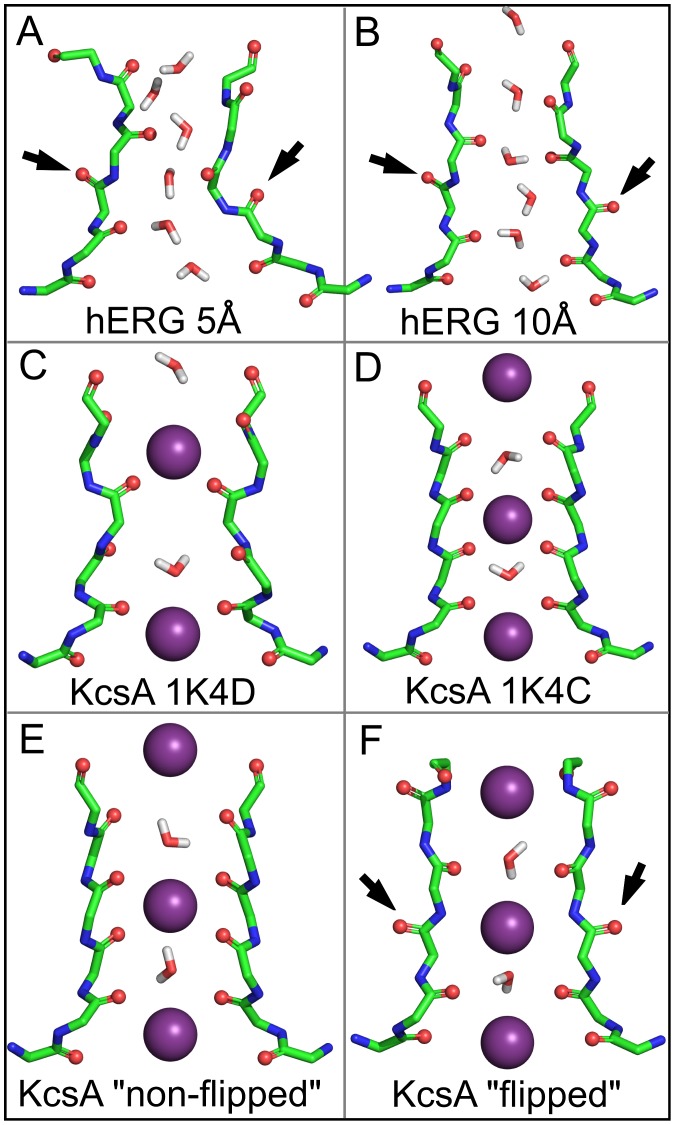
Selectivity filter conformations of hERG simulations and KcsA crystal structures. For clarity, only two subunits are shown. Snapshots were taken at the end of the simulations with a 5 Å N629–S620 distance (**A**), and a 10 Å N629–S620 distance (**B**). A flip of the V625 carbonyl group is seen (black arrows). For comparison, (**C**) displays the crystal structures of the collapsed (pdb: 1K4D) and (**D**) the conductive KcsA SF (pdb: 1K4C). (**E**) Comparison with the non-flipped (pdb: 1ZWI) and (**F**) flipped SF conformation (pdb: 2ATK) observed in crystal structures of the non-inactivating KcsA mutant E71A [8].

Fig. 2 displays a direct view of the SF observed in the two extremes of the distance scan with water molecules present at that particular time frame, together with a comparison to KcsA SF crystal structures. Please note that the transient presence of water does not imply stable binding sites. At the end of the d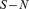 = 10 Å simulation, the SF backbone (Fig. 2B) had remained close to the configuration of the high K^+^ state (1K4C, Fig. 2D). This is remarkable, as K^+^ ions were not bound to the SF and the fluctuation level at the simulation temperature was relatively high. The only exception are transiently flipped carbonyl groups at V625, as previously reported (see below). By contrast, a distance d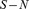 of 5 Å resulted in filter collapse toward a final state closely resembling the KcsA SF low-K^+^ state, again within the limits of thermal fluctuation (Fig. 2A,C). Please note that Fig. 2A,B show simulation snapshots at T = 310 K, and so, in contrast to crystals, a complete four-fold symmetry between the channel subunits cannot be expected.

The carbonyl groups of V625 were frequently observed to transiently flip backward (black arrows in Fig. 2A,B) in our simulations. In the past, such flips have been linked to non-conductive SF states (e.g. [6], [32]), but crystal structures and electrophysiological measurements of a non-inactivatable KcsA mutant (E71A, Fig. 2F) have demonstrated that flipped states of V625 retain a conductive channel [8]. In addition, recent crystallographic studies on the E71A KcsA mutant have concluded that ‘the flipped SF conformation is actually yet another conductive filter state, encountered with high frequency’ [34].

Taken together, our simulations suggest that the conformational state(s) of S620 and N629 control inactivation of hERG, which comprises a change of the hERG SF from a conductive conformation to a geometry resembling a collapsed SF state. From simulations with varying ion occupancy in the SF (Fig. S1), we conclude that the complete transition of the SF is promoted by at least a transient phase in which K^+^ ions are not bound in the filter, although the final conformation of the collapsed state exhibits a single K^+^ ion in the crystal structures [27]. In a range of further simulations, raised K^+^ occupancy disfavored collapse of the SF (Fig. S1). It is however important to note the limitations of the present hERG model, which in particular include the absence of the extracellular turret loop section which may contribute to inactivation.

### Alternating Conformations of N629 Act as Switch for the Selectivity Filter

We further examined the conformational toggle between S620 and N629 by investigating distances between d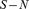 = 5–12 Å. The end points of six additional 50-ns MD simulations (d = 6,7,8,9,11 and 12 Å), together with the eight simulations for 5 and 10 Å, were determined by averaging the projection of the simulation trajectories onto the difference vector after 40 ns, as described above. As shown in Fig. 1D, the degree to which the SF was driven toward the collapsed state showed a strong dependence on d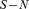. The relationship was found to be almost linear between d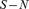 = 5–11 Å. Inter-side chain distances of 10 Å and 11 Å stabilized the highly ordered, conductive filter configuration, while small d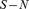 drove the SF toward the collapsed state. A sharp move toward the collapsed state was recorded at a distance of 12 Å (and larger distances, not shown). This demonstrates that the distance between S620 and N629 acts as a direct and nearly linear switch that can toggle the SF between its conductive and collapsed state. We identified the close interaction between S620 and N629 as a bidentate hydrogen bond that is stably formed around d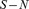 = 5 Å, and disrupted toward d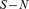 = 10 Å.

To test the behavior of known inactivation-deficient mutants of hERG in the light of these findings, we investigated both the mutant S620T [21] and the G628C/S631C double mutant [13], [21], [32], two intensely studied, non-inactivating forms of hERG. In the S620T mutant, a methyl group is added directly behind the SF on the acceptor residue of the proposed hydrogen bond toggle, providing more steric bulk which may alter a potential hydrogen bond network. In the double mutant G628C/S631C, an intra-subunit disulfide bond between sequence positions 628 and 631 is introduced, which is expected to enclose N629 and so to disrupt a possible hydrogen bond between S620 and N629 [32].

Interestingly, we did not observe a complete transition toward the collapsed state in the case of the S620T mutant, despite our simulation settings strongly imposing a distance of 5 Å between T620 and N629, which is the most favorable contact distance for collapse in the wildtype (Fig. 1C, magenta curve and Fig. 1D, magenta circle). This was due to the additional methyl group, which disrupted a close hydrogen bond contact there. In the G628C/S631C double mutant, the steric hindrance introduced by the disulfide bond inhibited such a close approach even further. Here, the simulations showed that d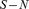 remained near 9 Å, a distance which stabilizes the SF near its conductive state (Fig. 1D). The mutant data shows that hERG variants with experimentally determined inactivation deficiencies also exhibit a changed behavior in our simulations. Both mutants were found to be incapable of fully reaching the collapsed SF state.

**Figure 3:**
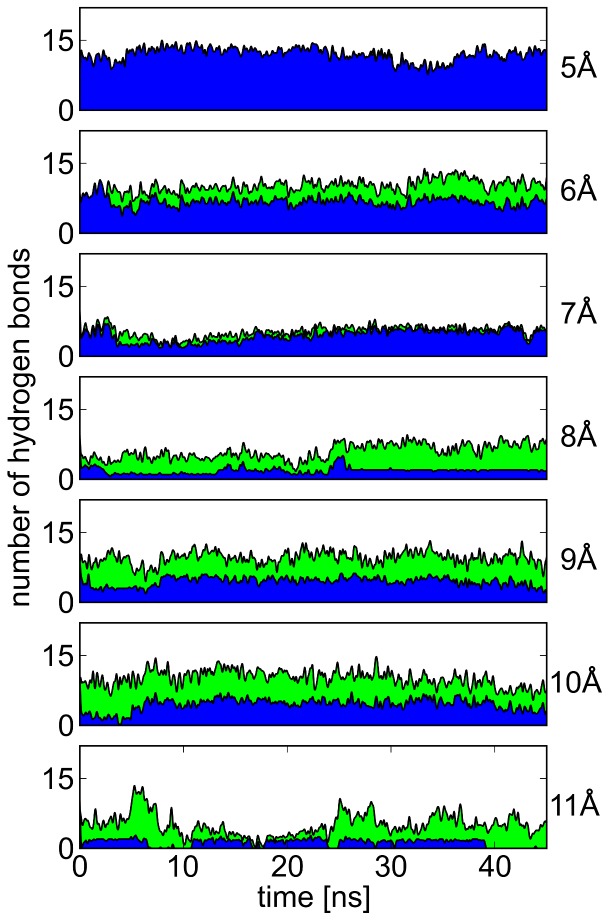
Hydrogen bonds formed by N629 at different S620–N629 distances. For each S620–N629 C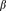 distance (right), the number of hydrogen bonds formed by the N629 side-chain to the same subunit (blue area, mainly S620) and to neighboring subunits (green area, mainly G628) is shown over simulation time. A steady rise in the proportion of inter-subunit hydrogen bonds can be seen with an increase in S-N distance.

To further evaluate the toggle function of N629 in the course of the simulations, we compared the number of H-bonds formed by N629 to the same and adjacent subunits, respectively (Fig. 3). At the smallest S–N-distance, N629 exclusively formed intra-chain H-bonds, whereas toward larger S–N distances, the proportion of H-bonds interconnecting neighboring subunits steadily increased. A closer inspection at S–N distances around 10 Å exhibited inter-subunit H-bonds formed between the side chain of N629 and the backbone carbonyl unit of G628 from the adjacent subunit as the dominant species (Fig. 1A). Such a connection can be conceived to stabilize the ordered state of the SF by confining the motion of the extracellular entry. Simulations of wt-hERG, in which inter-chain N629–G628 H-bonds were further stabilized by introducing a weak harmonic potential, showed that the SF continuously remained near its conductive conformation there, while the S620–N629 distance was around 9 Å such as in the G628C/S631C mutant (Fig. 1D).

### Experimental Evidence for the Role of N629 and a Neighboring H-bond Network

A number of mutations around the SF have been found to strongly affect C-type inactivation in hERG [18]. Furthermore, it could be demonstrated in KcsA that mutation of the H-bonding power of W67 strongly influenced KcsA inactivation and the strength of the E71–D80 interaction [35]. According to our structural model and simulations of hERG, the N629 side-chain forms the central inactivation switch by tightly interacting with S620.

**Figure 4:**
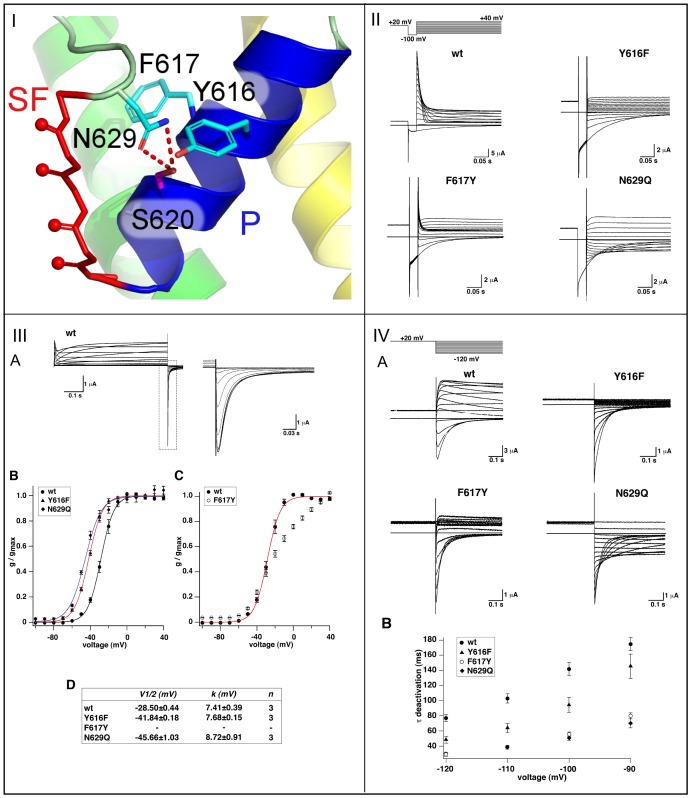
Mutations of the H-bond network behind the selectivity filter. (**I**) Mutation sites N629Q (pore loop), F617Y, and Y616F (both: pore helix, P). (**II**) Inactivation properties of wild-type and mutant hERG channels. Inactivation time courses for the different hERG channels were recorded as shown. A conditioning pulse to +20 mV followed by a 100 ms hyperpolarizing pulse to −100 mV preceded various depolarizing pulses from −90 to +40 mV in 10 mV increments as illustrated by the pulse protocol on top. (**III**) (A) Exemplary wild-type (WT) hERG current traces elicited by 6 s depolarizing voltage steps from −100 to +40 mV followed by a hyperpolarizing pulse to −140 mV. Respective tail currents are shown enlarged at left. (B) Conductance-voltage relations determined from Boltzmann fits to normalized tail current amplitudes for hERG wild-type and Y616F and N629Q mutant channels. (C) Conductance-voltage relation for the mutant hERG channel F617Y. (D) 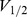 and *k* parameters obtained under steady state conditions from the Boltzmann fits for wild-type and mutant channels are summarized at the bottom. *p<0.05 versus wild-type. (**IV**) (A) Deactivation time courses of wild-type and mutant hERG channels. Tail currents were elicited according to the pulse protocol shown on top. (B) Voltage dependence of mean deactivation time constants (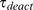) (n = 4) for the different channels as indicated.

We next aimed for independent experimental tests of these hypotheses. Therefore, we measured channel current of a mutant that directly altered the S620–N629 interaction by introducing a longer side-chain into this position (N629Q). Moreover, in our model, the S620–N629 link is surrounded by side chains that are likely to modulate the strength of an in-lying H-bond, either by their hydrophobicity or alternative H-bonding potential. Thus we additionally tested the conservative mutations Y616F, homologous to KcsA W67, and F617Y, which both comprise an altered H-bonding potential in its close vicinity. The effects on hERG inactivation were then monitored using whole-cell electrophysiology in the Xenopus oocyte expression system. Fortunately, the three mutant hERG channels expressed functional currents amenable to characterizing the mutational influence on hERG channel activation, deactivation and inactivation, respectively. The voltage dependence of hERG channel steady-state activation was measured with long duration (6 s) depolarizing test pulses. From a holding potential of −100 mV, test pulses ranged from −90 to +40 mV in 10 mV increments, followed by a hyperpolarizing test pulse to −140 mV for recording tail currents (Fig. 4, panel III). Tail current amplitudes were normalized and plotted against test potential (Fig. 4 III B, C). In agreement with previous data [36], Boltzmann fits to the data showed that the voltage of half-maximal activation (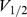) of wild-type hERG channels was −28.5 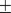 0.44 mV (n = 3) with a slope factor *k* = 7.41 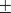 0.39 mV (n = 3). Activation of the mutant Y616F and N629Q hERG channels was shifted by 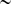
15 mV to more negative potentials in comparison to wild-type (see Fig. 4, panel III B). The steady-state values of V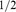 and *k* of wild-type and mutant hERG channels are summarized at the bottom of Fig. 4 III. Because the current-voltage relations for the F617Y mutant hERG channel apparently showed two phases, we did not fit a Boltzmann function to the F617Y data (Fig. 4 III C).

Deactivation time courses were recorded by applying a depolarizing conditioning pulse to +20 mV for 1.6 s from a holding potential of −100 mV, followed by various test potentials from −120 to +50 mV in 10 mV increments for 6 s. Single exponential fits to the data were used to obtain deactivation time constants (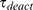; Fig. 4 IV). Consistent with previous data [20], 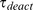 was, at −120 mV, 73.2 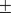 4.1 ms (n = 7) for wild-type hERG and, on average, 33.7 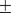 3.2 ms (n = 6–9 for each mutant) for the different mutant hERG channels (Fig. 4 IV). In comparison, time rise to peak at +20 mV was not markedly different between wild-type and mutant hERG channels (on average 59.1 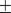 1.8 ms; n = 3–4 for each channel).

The three mutations however had a dramatic effect on hERG channel inactivation (Fig. 4 II). In contrast to wild-type, which inactivated rapidly, the mutant N629Q hERG channels were devoid of inactivation (n = 8). In the case of Y616F and F617Y hERG channels, we observed a strong inward rectification (Fig. S2). It indicated that the mutations had shifted the voltage-dependence of hERG steady-state inactivation to very negative test potentials. Steady-state inactivation was determined at different test potentials from the ratio of instantaneous current amplitude and current amplitude remaining 100 ms after the onset of the test potential. We plotted the normalized inactivation data as a function of voltage (Fig. S2). Fitting a Boltzmann function to the wild-type data, we estimated that the voltage of wild-type half-maximal steady-state inactivation (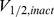) was −18.8 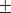 0.8 mV (n = 3; S.E.M.), in agreement with data in the literature [37], [38]. 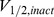 for the Y616F mutant was so strongly negatively-shifted that we were unable to obtain sufficient data points for measurement. 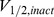 for the F617Y hERG mutant was negatively-shifted by about 100 mV. Using an open fit to our limited set of data we estimated 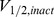 for the Y616F hERG channel at −126.6 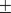 1.3 mV (n = 3; S.E.M.). Also, the F617Y mutation affected the inactivation time course (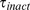). Fitting a single exponential to the inactivation time courses, we obtained at −20 mV for the wild-type channel a 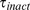-value of 21.73 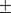 1.63 ms (n = 4) and for the F617Y mutant one of 5.57 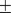 0.25 ms (n = 4; Fig. 4 II).

### Structural Links to Modulation of Cavity Shape and Effect of hERG Agonists

Impairment of C-type inactivation is responsible for short-QT syndrome and plays a key role in drug-induced gain of function in hERG [23]. hERG agonists are of great potential therapeutic interest [39], [40], forming a possible basis for treatment of patients suffering from inherited long-QT hERG mutations [23]. We were therefore interested in the interplay between small molecule binding to the inner cavity of the hERG channel and C-type inactivation.

We focused on two recently described hERG agonists, PD-118057[23] and ICA-105574 [41], [42]. Both molecules have been shown to interact predominantly with a hydrophobic binding pocket near residue F619 on the hERG pore helix [23], [42]. On the basis of our previous results, the inactivated state of hERG was modelled by simulating a conformation with a collapsed SF, while the open form was assumed to exhibit a conductive SF. In both cases, we carried out 100-ns MD simulations.

**Figure 5:**
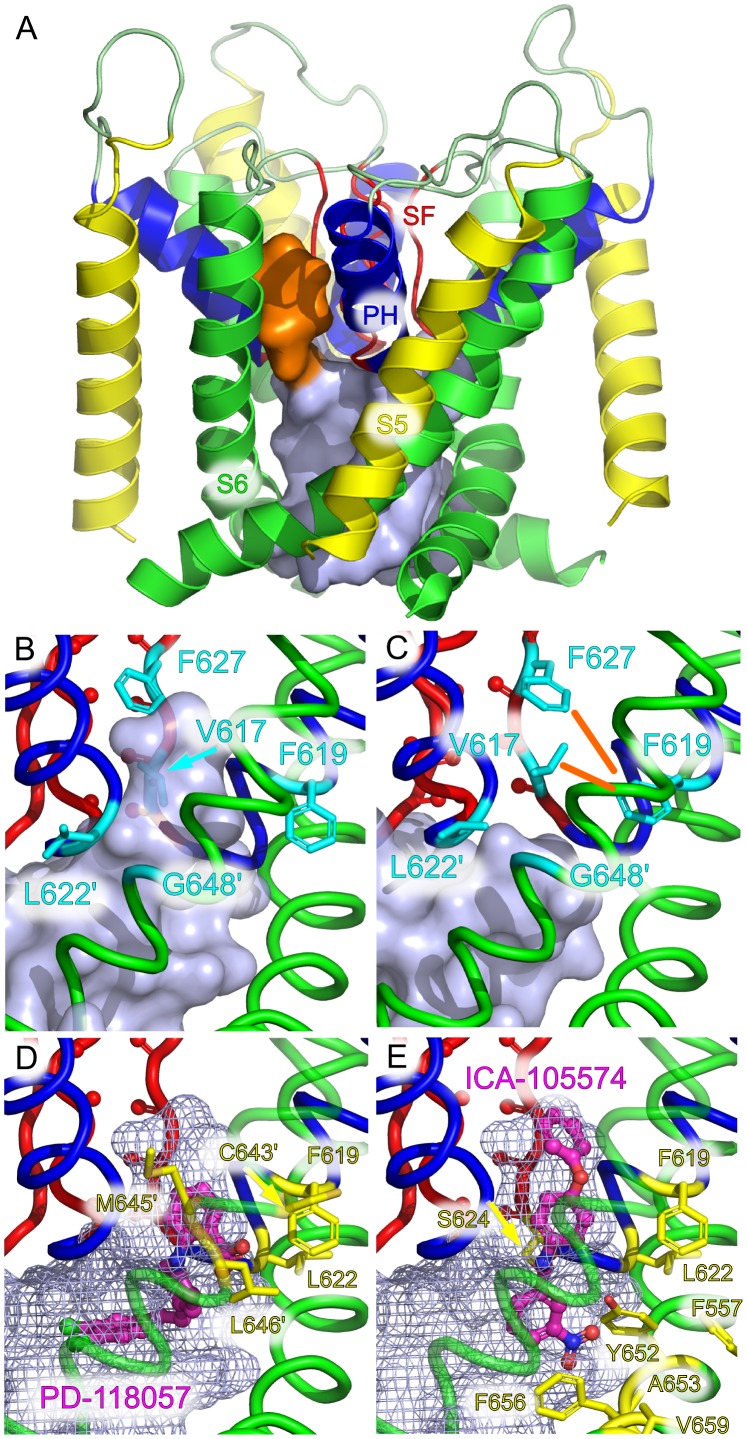
Suggested mechanism of action for hERG activators. (**A**) Binding pocket for activators, shown here located between the pore helices of two adjacent subunits (orange surface). (**B**) The experimentally determined binding pocket for PD-118057 and ICA-105574 is located around residue F619 and extends to residue L622 (secondary subunit contacts are marked with a prime symbol). (**C**) A cascade of conformational changes triggered by collapse of the SF leads to constriction of the binding pocket (orange lines) and rearrangement of L622. (**D**) The cavity is large enough to accommodate PD-118057. All residues known to affect PD-118057 binding [23] line the pocket (yellow). (**E**) The activator molecule ICA-105574, shown docked to the side-pocket with residues known to influence binding in yellow.

The conformational change at the SF initially had a moderate direct spatial extent (initial RMSD in the SF: 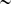
1 Å). Intriguingly however, it had far-ranging subsequent consequences near the pore helix and the interface with S6 in the trajectories: The subtle conformational change within the backbone of the SF (S624-G628) was gradually amplified by inducing side chain rotations, in particular those of V625 and F627. This reordering led to rotation of the directly neighboring residue L622 on the pore helix and the main drug binding site F619, one helical turn upward. In concert, these rearrangements in the activated open state were found to be capable of opening a side pocket, extending from the main cavity (Fig. 5 A,B), which was found to be wide enough to accommodate either PD-118057 and ICA-105574 in molecular docking calculations. In contrast, the pocket was smaller and only transiently present in the inactivated state, blocking their entry (Fig. 5 C).

A closer look at the residues found to be important for activator interaction reveals that both the location and extent of the side-pocket are in good agreement with the main interaction sites identified in Refs. [23] and [42] (Fig. 5. D,E). The residues shown to interact with ICA-105574 are however located at the lower entrance of the side-pocket and so a slightly different mode of binding is suggested (Fig. 5E). We propose a mechanism, in which activator molecules belonging to this class are able to bind in this side-pocket in the activated open, but not in the inactivated state. Thus, by stabilizing the activated state, inactivation is putatively impaired by these molecules.

## Discussion

Inactivation of hERG is of crucial physiological and medical importance, as it is instrumental in controlling the duration of the cardiac action potential. Our simulations suggest that the inactivated state of hERG resembles a collapsed SF conformation and propose a mechanism for hERG entry into inactivation, relying on the group N629 alternating between intra- and inter-subunit hydrogen bonds as a toggle. The toggle is influenced by a finely-tuned neighboring H-bond network. Non-inactivating mutants are observed to be unable to form an intra-subunit H-bond conformation and thus do not reach a collapsed SF state. To validate these findings, we conducted mutation studies and electrophysiology experiments which showed a dramatic effect of conservative mutations that alter the steric properties and H-bonding potential near the S620-N629 switch.

Recently, it has been shown that a similar collapse of the SF likely underlies slow inactivation in the prokaryotic K^+^ channel KcsA, with a network of H-bonds around D80 controlling the state of the SF [4], [35]. There are important functional differences between slow inactivation in KcsA and hERG inactivation. However, recent mutational studies highlight the mechanistic similarities between KcsA and Kv channel inactivation, given the high level of functional and structural conservation in the K^+^ channel pore domain [4], [30]. Our work thus expands the findings on KcsA into the domain of voltage-gated K^+^ channels. The tight interaction between two side chains in the same subunit that form a strong H-bond (S620 and N629) may explain the strong inactivation tendency of hERG as compared to other K^+^ channels, where the homologous groups have a weaker H-bonding potential (e.g. Kv1.3, KcsA). The hydrophobic character of the immediate environment, possibly also the unusual sequence of the hERG SF (GFG instead of GYG), contribute to the strength of this H-bond and the rapid time course of hERG inactivation. Our results also have some bearing on a recent electrophysiological study on Kir1.1 [43], which indicated an inter-subunit salt bridge network strongly affecting inactivation of the inwardly rectifying Kir1.1 channel. Kir1.1 residues involved in these salt bridges (E118, R128, and E132) occupy sequence positions homologous to S620, V630, and T634 in hERG, suggesting a similar role of an inter−/intra-chain H-bond equilibrium controlling inactivation.

We suggest that the S620-N629 interaction constitutes the innermost element of a wide range of allosteric switches that can alter inactivation in hERG and the stability of the open state of the channel. Some of the wide-ranging rearrangements were elegantly demonstrated by Wang et al. [30]. More distant effects include sequence-distal interactions such as S641, mutations of which have been demonstrated to impact inactivation [30], [44] and, possibly, domain motions encompassing regions as far as helices S6, S4, and the S4–S5 linker in early stages of inactivation [30]. In line with this, our mutations slightly affect hERG current-voltage-relations and deactivation time-courses. Our study shows an intricate network of hydrogen bonds that may have a profound effect on the workings of the inner switch and on the link between inactivation and activation gating [45].

The intensely studied mutant hERG channel hERG S620T and the double mutant G628C/S631C, which have shown inactivation-deficiency in earlier experimental studies [13], [21], [32], were investigated and displayed an inability to attain a collapsed SF state. The importance of the proposed switch may also become evident from comparison with the closely related hEAG1 channel, in which threonine occupies the location homologous to S620 in hERG (T432 in hEAG). Similar to the hERG S620T mutant, hEAG lacks C-type inactivation [46].

Unfortunately, a complete structural model of hERG including the turret loop region is presently not available, owing to its largely disordered character. Our investigation relies on the present consensus hERG model [28], such that more distant interactions including the turret section were not addressed here. Most cases of inherited short-QT syndrome are however elicited by the hERG mutation N588K in this turret region [24]–[26]. Sequence position 588 is located in a stretch of residues characterized by the fact that each group strongly affects inactivation when it is mutated (W585–G594) [18]. It had hence been suggested and later confirmed that this stretch forms an alpha-helical section within the turret loop, which immediately borders the SF [47], [48]. According to these data, N588 is located directly next to the SF mouth and thus to N629. We speculate that a lysine at this position could either interact unfavorably with the H-bonding network that controls inactivation or act like SF-binding lysines in channel toxins, which have been shown to induce recovery from inactivation in K^+^ channels by an interaction near the filter [7].

On a longer range, we found that the conformational change of the SF from the collapsed to the conductive state altered the shape of the hERG inner cavity. Our simulations showed that the hERG agonists PD-118057 and ICA-105574 are bound in a side pocket of the cavity near F619 that is large enough to accommodate the agonists, but only exists in the activated state. It had been previously established by experiment that PD-118057 and ICA-105574, drugs that inhibits inactivation and thus increase hERG current, bind near F619 and L622 [23], [42]. Such hERG agonists are considered a new approach for the treatment of long-QT associated arrhythmia. Our results provide a simple explanation for the activation of hERG by these agonists, which may also hold true for other hERG activators: The groups V625 and F627, part of the SF, are prevented from adopting their collapsed-SF positions by the action of this small molecule (Fig. 5B,C), i.e. the SF cannot reach its inactivated conformation. In contrast to hERG blockers which bind inside the ion conduction pathway and reduce current [10], binding of activator molecules to the side-pocket does not negatively affect ion flux through the pore [23].

## Methods

### Molecular Dynamics Simulations and Docking

All simulations were based on the recent consensus homology model of the pore-forming region of hERG, comprising helices S5 and S6, the pore helix, and the pore loop [28]. All simulations were carried out with the Gromacs simulation software, version 4.0 [49]. The hERG model was inserted into a simulation box with a fully hydrated and equilibrated membrane consisting of 176 dimyristoylphosphatidylcholine (DMPC) molecules and 12 888 water molecules using g_membed [50]. The amber99sb force field was used for the protein and ions [51]; parameters for DMPC were derived from Berger et al. [52] and the solvent was modeled using the SPC/E water model [53]. Electrostatic interactions were calculated explicitly at a distance smaller than 1.0 nm, long-range electrostatic interactions were treated by particle-mesh Ewald summation at every step [54]. Lennard-Jones interactions were calculated using a cut-off of 1.0 nm. The LINCS algorithm was employed to constrain all bonds [55], allowing for an integration time step of 2 fs. The simulation temperature was kept constant by weakly (t = 0.1 ps) coupling the lipids, protein and solvent separately to a temperature bath of 310 K by using the velocity-rescale method [56]. Likewise, the pressure was kept constant by Berendsen coupling of the system to a pressure bath of 1 bar [57]. To ensure a conductive conformation of the SF at the beginning of the simulations, the system was equilibrated for 1 ns with position restraints on the SF backbone atoms using the high-[K^+^] crystal structure of KcsA as reference (PDB entry 1K4C [27]) with a force constant of 1000 kJ mol^−1^ nm^−2^. For the S620–N629 distance scan simulations, a distance restraint between the C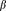 atoms of S620 and N629 was introduced by means of a harmonic potential with a force constant of 5000 kJ mol^−1^ nm^−2^. In simulations of the collapsed SF, the low-[K^+^] conformation of the SF was used together with position restraints of 1000 kJ mol^−1^ nm^−2^. After energy minimization and equilibration of the system using position restraints of the same strength on all protein heavy atoms for 2 ns, simulations of 50–100 ns length were carried out. The collective coordinate describing the transition between the high- and the low-[K^+^] crystal structures of the KcsA SF was obtained by performing a principal component analysis of the SF backbone on the set of PDB entries 1K4C and 1K4D [27]. To derive the data points shown in Fig. 1D, we used the average projection of each simulation on the difference vector after 40 ns. Dockings of PD-118057 [23] and ICA-105574 [42] were performed using FlexX from LeadIT [58] with standard parameters. A degree of flexibility of protein side chains was introduced as derived from the simulations. The spatial extent of the side-pocket was determined by calculating its solvent accessibility for molecules the size of water using in-house code. Independently, the accessibility of the pocket for PD-118057 and ICA-105574 molecules was investigated by using FlexX. The receptor surface was defined using a radius of 18 Å around S620 nearest to the side-pocket. The best scoring molecule docked in the side-pocket was selected for visual representation.

### In vitro Transcription and Functional Expression in *Xenopus laevis* Oocytes

mRNAs were prepared from hERG wild-type or mutant constructs (Y616F, F617Y and N629Q) in pGEMHE using the mMESSAGE mMACHINE T7 kit (Ambion) according to the manufacturer's instructions. Oocyte incubation and cRNA injections were performed as described previously [59]. Briefly, 1 day after surgery, oocytes were injected with cRNA (1–2 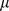
g 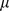
l^−1^). Functional expression was typically assessed 1–3 days after microinjection. Inward current levels were in the range of 1–10 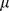
A at repolarizing voltages of −100 mV to ensure proper voltage control.

### Electrophysiological Recordings

Whole-cell currents were recorded under two-electrode voltage control using an Oocyte-clamp amplifier (Oc-725C Oocyte-clamp, Warner Instrument Corp., USA). Glass microelectrodes (World Precision Instruments, Sarasota, FL, USA) were pulled (DMZ Universal puller, Zeitz Instruments, Martinsried, Germany), and their tips were bevelled (Micro forge, Narishige Co. LTD, Tokyo, Japan) to obtain resistances between 0.1–2 M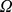. The pipettes were filled with 3 M KCl and the oocytes were superfused with ND96 solution: (in mM) KCl 2, NaCl 96, CaCl_2_ 1, MgCl_2_ 1, HEPES 5, pH 7.4 (adjusted with 1 N NaOH). All currents were digitally sampled at 2 kHz and leak and capacitive currents were corrected on-line using the P/4 subtraction method. The sweep interval was 25 s and the holding potential was −100 mV. Data were converted with an AD/DA-computer interface (TIC16, Instrutec Corporation, Great Neck, USA) and stored on a personal computer. All experiments were performed at room temperature (20–23°C). The program packages Pulse+Pulsefit (HEKA Elektronik, Lambrecht, Germany) and IGOR Pro (WaveMetrics Inc., Oregon, USA) were used for data acquisition and analysis.

### Experimental Data Analysis

The voltage dependence of activation was assessed by standard tail current analysis using repolarizing pulses to −140 mV. Tail current amplitudes were normalized to maximum. A Boltzmann function 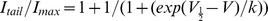 was fit to the data to estimate 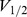 and the rate constant *k*, where 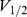 is the potential at half-maximal current activation and *k* the slope factor. A single exponential function was fit to the deactivating currents to using Pulsefit software. Steady-state inactivation was determined essentially as described by Zhang et al. [38]. The ratio of instantaneous current amplitude and amplitude of current remaining after 100 ms was taken as measure of steady-state inactivation at a given test potential. The voltage of half-maximal steady-state inactivation (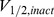) was calculated by fitting a Boltzmann function to the data. To obtain an estimate of 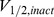 for F617Y hERG channels, we used an open fit procedure with the assumption that the mutation did not alter the slope of the steady-state inactivation–voltage relation. Data are given as mean 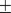 S.E.M.

## Supporting Information

Figure S1 **Distance scan (S620-N629) with a single K^+^ ion in the hERG SF.** Position on the interpolation vector at the end of the simulations. In all cases in which the ion remained in the SF (red dots), the conformation of the SF remained stable in an intermediate state between the “ordered” 1K4C and “collapsed” 1K4D conformation. At Cβ distances of 6 and 8 Å, the K^+^ ion diffused out of the channel (green dots) resulting in a conformational change of the selectivity filter toward the collapsed state. This effect is more pronounced at shorter S-N distance. We ascribe the lesser extent of the transition toward a collapsed state to a partial inhibition caused by the presence of ions in the SF. In our interpretation of the results, a complete SF transition requires at least a transient vacation of the SF, in spite of the fact that the final state displays the presence of a single ion in the crystal structures. The final state may then become reoccupied with one K^+^ ion on longer timescales.
(TIF)

Figure S2 **Wild-type and mutant hERG inactivation.** (A) Steady-state inactivation plotted against the voltage of the test pulse. For experimental details see Methods. Solid lines correspond to a Boltzmann fit to the data (n = 3; S.E.M.). (B) Voltage-dependence of tail-current amplitudes. Data were obtained using the pulse protocol shown in Figure 4 (IV).
(TIF)
