# Supplementary figures and images for: A Molecular Switch Driving Inactivation in the Cardiac K+ Channel hERG
Source: PLoS One. 2012 Jul 24;7(7):e41023. doi: 10.1371/journal.pone.0041023 (PMC3404103; doi:10.1371/journal.pone.0041023)

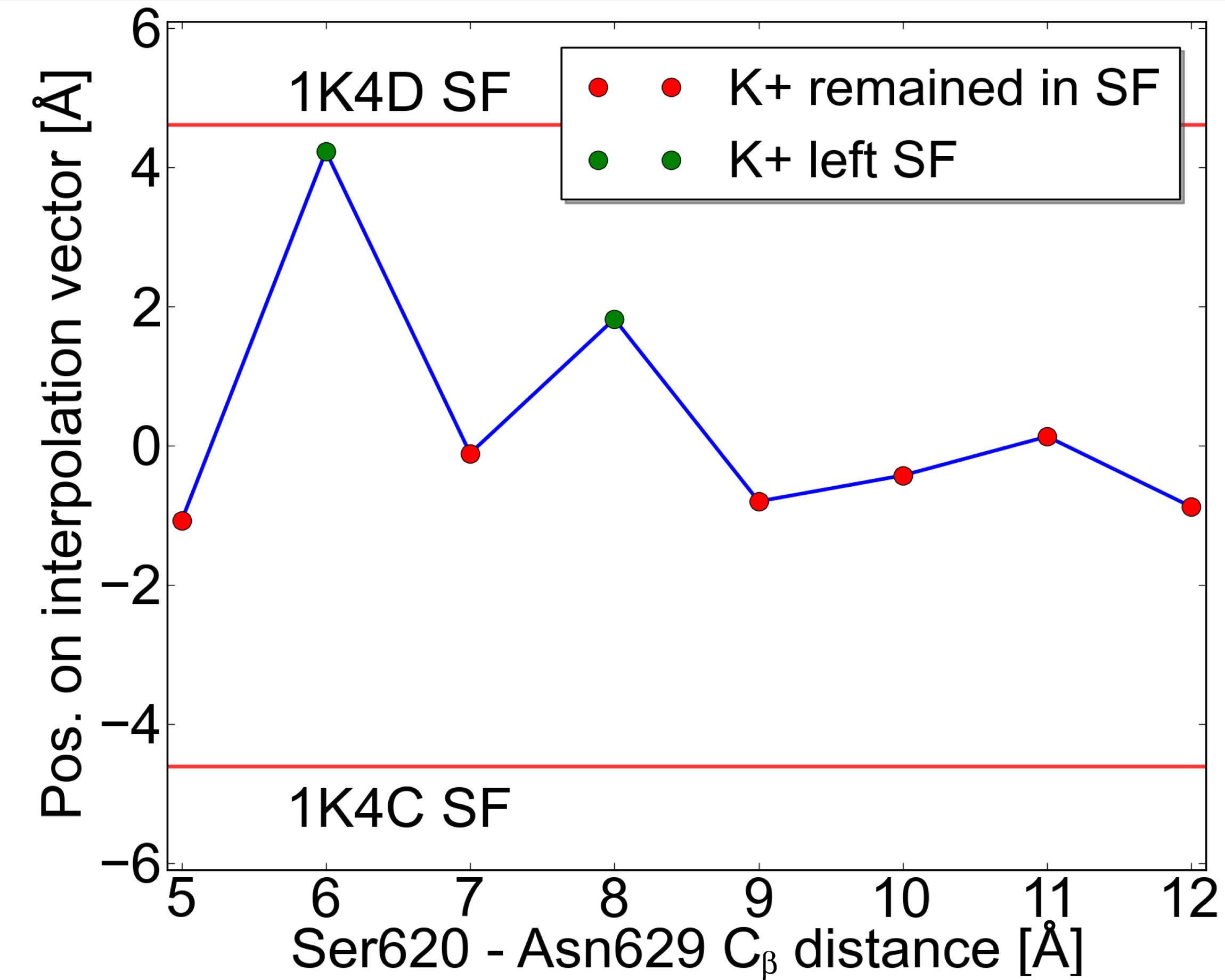

Supplement: Figure S1 — Distance scan (S620-N629) with a single K+ ion in the hERG SF. Position on the interpolation vector at the end of the simulations. In all cases in which the ion remained in the SF (red dots), the conformation of the SF remained stable in an intermediate state between the “ordered” 1K4C and “collapsed” 1K4D conformation. At Cβ distances of 6 and 8 Å, the K+ ion diffused out of the channel (green dots) resulting in a conformational change of the selectivity filter toward the collapsed state. This effect is more pronounced at shorter S-N distance. We ascribe the lesser extent of the transition toward a collapsed state to a partial inhibition caused by the presence of ions in the SF. In our interpretation of the results, a complete SF transition requires at least a transient vacation of the SF, in spite of the fact that the final state displays the presence of a single ion in the crystal structures. The final state may then become reoccupied with one K+ ion on longer timescales. (TIF) [file pone.0041023.s001.tif]

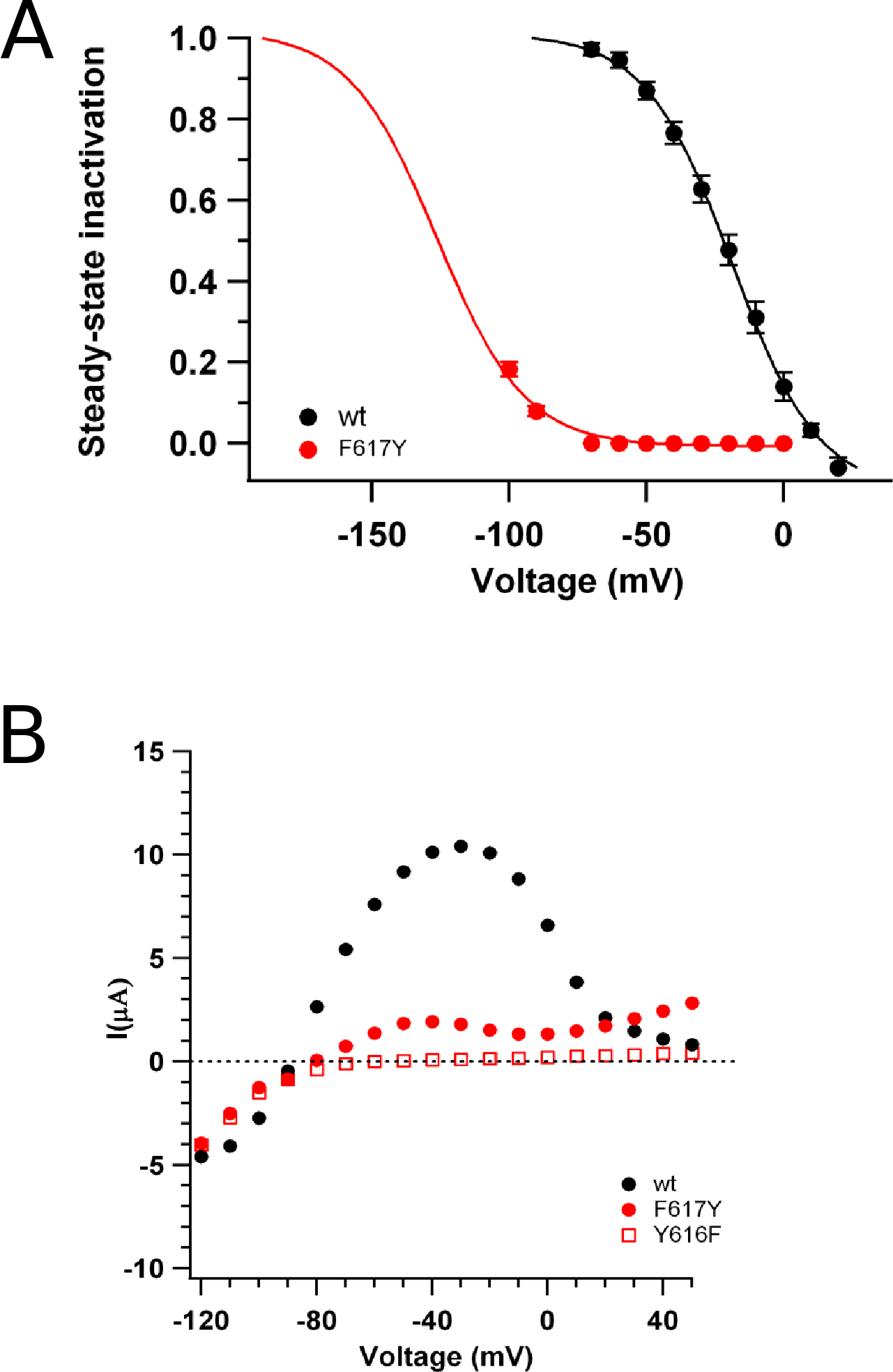

Supplement: Figure S2 — Wild-type and mutant hERG inactivation. (A) Steady-state inactivation plotted against the voltage of the test pulse. For experimental details see Methods. Solid lines correspond to a Boltzmann fit to the data (n = 3; S.E.M.). (B) Voltage-dependence of tail-current amplitudes. Data were obtained using the pulse protocol shown in Figure 4 (IV). (TIF) [file pone.0041023.s002.tif]
